# Marital satisfaction in Iranian infertile women: A systematic review and meta-analysis

**DOI:** 10.3389/fpubh.2022.1027005

**Published:** 2022-11-25

**Authors:** Amir Tabatabaee, Arezoo Fallahi, Bahre Shakeri, Vajiheh Baghi, Reza Ghanei Gheshlagh

**Affiliations:** ^1^Department of Nursing, Islamic Azad University, Quchan Branch, Quchan, Iran; ^2^Social Determinants of Health Research Center, Research Institute for Health Development, Kurdistan University of Medical Sciences, Sanandaj, Iran; ^3^Faculty of Nursing and Midwifery, Kurdistan University of Medical Sciences, Sanandaj, Iran; ^4^Besat Hospital, Kurdistan University of Medical Sciences, Sanandaj, Iran

**Keywords:** marital satisfaction, infertility, systematic review, infertile women, marriage

## Abstract

**Background:**

Infertility is a severe crisis in life that, in addition to creating psychological problems and disrupting a parent's identity and role, negatively impacts couples' marital satisfaction. Different studies in this field have reported different results, so this systematic review and meta-analysis was conducted to estimate the marital satisfaction standard score among infertile Iranian women.

**Method:**

The databases of PubMed, Scopus, Web of Science, Embase, Scientific Information Database, and MagIran were searched without a time limit. We used the meta-analysis and a random-effects model to estimate the marital satisfaction pooled score. The heterogeneity of studies was examined with the *I*^2^ index and Cochrane's Q test. The correlation between the pooled score with the publication year and the mean age of women was evaluated using meta-regression. We assessed the publication bias by the Egger test.

**Results:**

Seventeen studies with a sample size of 2,421 people were analyzed. The marital satisfaction pooled score of infertile women was 49% (95% CI: 39–60%). The marital satisfaction score in region 1 of the country (54, 95% CI: 42.7–65.3%) was higher than in other regions (45, 95% CI: 40–58%). Also, the marital satisfaction pooled score based on the Enrich scale (54, 95% CI: 39–69%) was higher than the score reported on other scales (45, 95% CI: 29–61%). Publication bias was not significant.

**Conclusion:**

Infertile women have moderate to low marital satisfaction, rooted in the culture and context of Iranian society. It seems necessary to provide measures to strengthen marital satisfaction, strengthen family relationships and prevent family disintegration in these women.

## Introduction

Pregnancy and motherhood are part of women's identity, and infertility as a stressful factor affects them emotionally and socially (1). From a clinical point of view, infertility refers to the absence of pregnancy after 12 months of regular and unprotected intercourse, although some couples get pregnant without treatment (2). The inability to have children is an obstacle to achieving the goal of parenthood, which changes the life of couples (3). Also, the stigma related to infertility in social interactions leads to a high level of internal distress that affects the marital relationship of couples (4). The quality of couples' relationships guarantees the stability of the family structure, and ineffective relationships and unsuccessful marriages threaten the mental health of couples and the survival of the family unit (5).

Marital satisfaction is an individual psychological state that reflects a marriage is perceived benefits and costs. The higher the cost, the lower the satisfaction with marriage and partner, and the higher the perceived benefits, the higher the satisfaction with marriage and life partner (6). Long-term research has shown that the satisfaction of newlyweds decreases with time, which is called the phenomenon of “honeymoon-is-over” (7) or the pattern of “honeymoon then years of blandness” (8, 9). Infertility makes couples experience these phenomena more intensely. Because having children in Iran is of great historical, religious, and cultural importance, infertility has become one of the reasons for family disputes, men's second marriages, domestic violence, and divorce. Although men and women play an equal role in infertility, it is considered a woman's problem in traditional societies. Hence, women are more responsible for infertility, even if the husband is the cause of infertility (1).

According to the World Health Organization (WHO) guidelines on the psycho-social aspects of infertility, healthcare workers must pay attention to the psychological aspects of fertility disorders and their promotion in addition to diagnosing and providing clinical interventions (10). Many studies in Iran have investigated marital satisfaction in infertile women and reported different results. In these studies, the standard score of marital satisfaction varied from 9.4 to 79.4% (11, 12). Therefore, it seems necessary to estimate the overall score of marital satisfaction in these women. Considering the status of the patriarchal and traditional society of Iran (13), knowing about the marital satisfaction of these women can help health officials in Iran to gain sufficient knowledge about this problem and take the necessary measures based on it.

This study aimed to estimate the marital satisfaction pooled standard score among Iranian women with infertility.

## Methods

This systematic review and meta-analysis study was conducted based on the Preferred Reporting Items for Systematic Reviews and Meta-Analysis (PRISMA) guidelines (14).

### Search strategy

The databases PubMed, ISI, Scopus, Embase, Google Scholar, Scientific Information Database (SID), and MagIran without a time limit were searched with the following keywords: “Marital status” OR “marital quality” OR “marital satisfaction” OR “marital adjustment” AND “infertility” OR “infertility^*^” OR “reproductive sterility” OR “subfertility” OR “sub-fertility” AND “Iran” OR “Islamic Republic of Iran.” The Farsi equivalent of these keywords was used in national databases. The search was conducted from the 8th to the 28th of July 2022.

### Data extraction

The articles that met the following criteria were included in this systematic review: (1) observational studies, (2) Providing a raw score of marital satisfaction based on standard scales, and (3) publishing articles in Farsi or English. In the studies conducted on infertile couples, and the marital satisfaction score was reported in two groups of women and men separately, we included the marital satisfaction score of infertile women in the analysis. Besides, the exclusion criteria were interventional, review, qualitative studies, case reports and letters to the editor, preprint studies, lack of access to the full text of articles, failure to report the raw score of marital satisfaction, and the report of marital satisfaction in the form of rank. The studies in which sufficient explanations regarding the measurement tool and scoring method were not provided were excluded from the analysis. After removing duplicate studies, the titles and abstracts of retrieved articles were independently reviewed by two authors. Then, the full text of the eligible studies was evaluated independently based on the inclusion and exclusion criteria by two other authors. In the next step, two other authors extracted the required information independently. A disagreement between the two authors was resolved by discussion. The necessary data, including the first author, the year of publication of the article, the location of the study, the sample size, the mean age of the women, and the type of instrument used to measure marital satisfaction, were recorded in the Excel form.

### Outcome

The outcome of the study was the marital satisfaction pooled score, which was measured by different scales such as Enrich, Nathan Azrin's marital satisfaction scale, and Index of Marital Satisfaction. Because different scales or versions of the same scale were used in these studies, and the number of items and their scoring were different, we converted all the reported raw scores into a standard score based on the following formula:


$$\begin{array}{l} \text{Transformed Scale}=\left[\frac{\left(\text{Actual raw score}\right)-\left(\text{lowest possible raw score}\right)}{\text{possible raw score range}}\right]\times \;100 \end{array}$$

The actual raw score presents the raw values acquired by summation, the lowest possible raw score donates the lowest possible raw value, and the possible raw score range shows the range between the highest and least raw scores that can be calculated (15).

### Quality assessment

To check the methodological quality of our articles, we used the Strengthening the reporting of observational studies in epidemiology (STROBE) checklist (16). Based on the objectives of the present study and the nature of the selected articles, we selected items from this checklist and evaluated the articles based on them. These items were: (1) Is the type of study specified in the title or abstract? (2) Are the objectives or hypotheses reported in the introduction? (3) Is the research environment defined? (4) Are the inclusion and exclusion criteria in the study defined? (5) Is the method of estimating the sample size mentioned? (6) Is the method of the statistical analysis reported? (7) Is the descriptive information of the studies reported? (8) Are the essential findings of the study discussed? (9) Are the limitations of the study mentioned? Moreover, (10) is the study's financial support source mentioned? If any of these items are mentioned in the article, that item gets a score of 1. Otherwise, it gets a score of zero. Based on this, the final score varies between 0 and 10, which were divided into three categories: < 4 (low quality), 4 to 7 (moderate quality), and more than 7 (high quality).

### Data analysis

A systematic analysis approach was used to the marital satisfaction pooled standard score in all eligible studies. In these studies, the available evidence is combined and evaluated to answer a specific research question. Random or fixed effects models were selected for analysis based on statistical tests for heterogeneity. Heterogeneity between studies was used using Cochran's Q test and *I*^2^ statistic, which indicates the percentage of changes between studies. Values of 25, 50, and 75% indicate low, moderate, and high heterogeneity, respectively. When the heterogeneity among studies is low and moderate (*I*^2^ < 50%), the fixed effects model is used; otherwise, the random effects model is used. To show the heterogeneity, we performed a subgroup analysis according to the region of the country and the type of scale. In addition, meta-regression was also performed to discover potential sources of anomalies. In subgroup analysis, all participants' data are divided into subgroups to compare them. Subgroup analyses may be performed as a tool to investigate heterogeneous outcomes or answer specific questions about specific patient groups, intervention types, or study types (17). In addition, meta-regression was also performed to discover potential sources of anomalies.

Meta-regression describes the linear relationship between (both continuous and categorical) study level covariates and the effect size (18). To evaluate the impact of each study on the pooled standard score, we used sensitivity analysis. In sensitivity analysis, the impact of individual studies on the final result is evaluated. If the results of one or more studies significantly change the final result, that result is not stable (19). In this study, publication bias was evaluated with Egger's Test. Publication bias occurs when the results of a study influence the decision to publish or distribute that study. Therefore, the publication of only the results that show a significant finding disturbs the balance of the findings in favor of positive results (20). All statistical analyzes were performed with Stata version 16.

## Results

### Description of included studies

**Figure 1:**
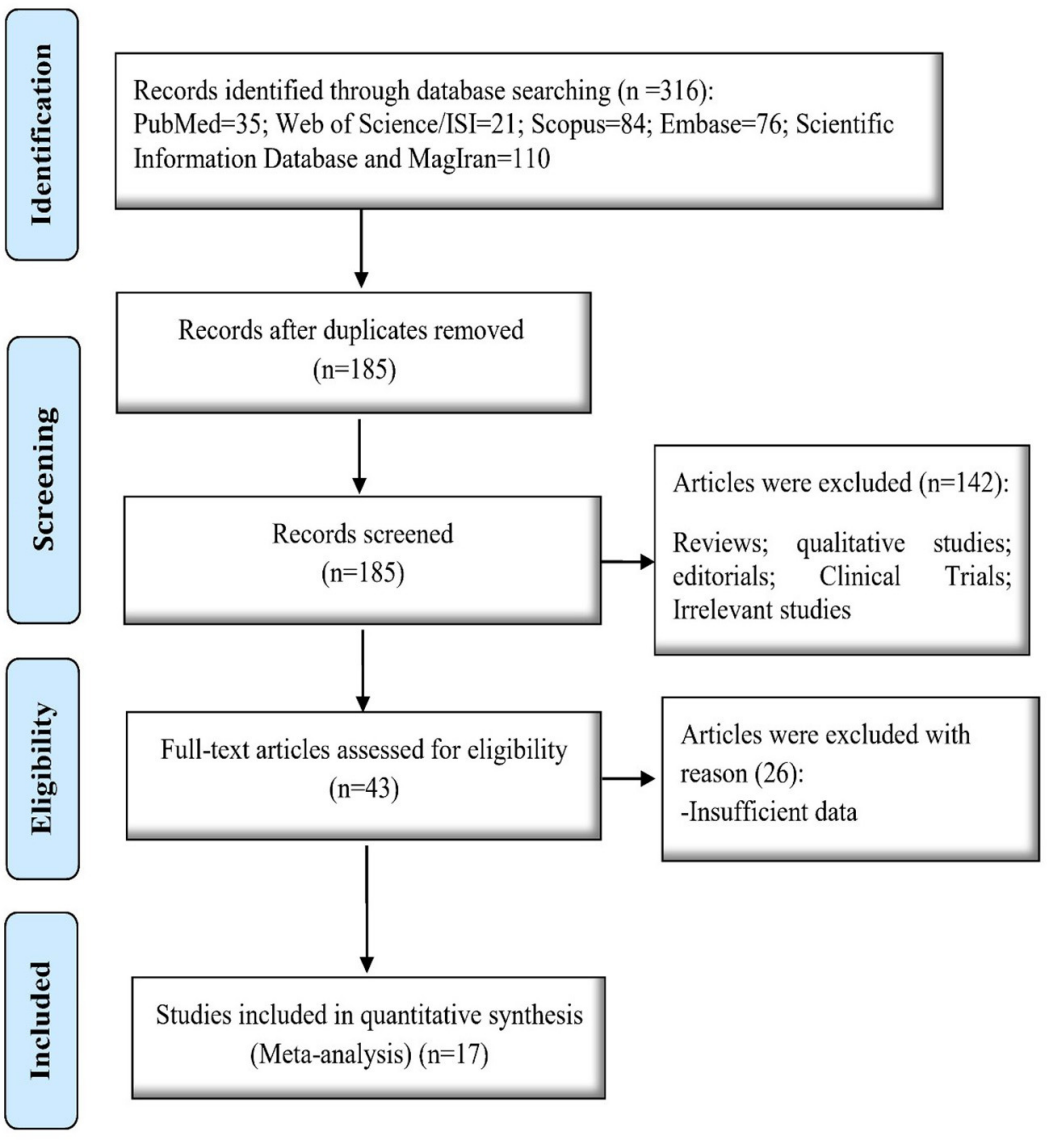
The screening process of selected articles.

By searching the mentioned national and international databases, 326 articles were retrieved, of which 141 were duplicates. In the next step, two authors independently read the title and abstract of the remaining articles. Based on the inclusion and exclusion criteria, 142 articles were excluded, and the full text of the remaining 43 articles was reviewed and evaluated. Marital satisfaction score was not reported in 26 studies, so we excluded these studies. Finally, we included 17 studies in the analysis (Figure 1).

### Characteristics of original articles

Eight studies were conducted in region one of the country (Tehran and its neighboring provinces), and the remaining nine studies were conducted in other regions of the country. In 15 studies, the marital satisfaction score was measured with the Enrich scale. Six studies had high methodological quality (21–26), and the rest were of moderate quality. The details of the selected articles are presented in Table 1.

**Table 1:** The characteristics of selected articles.

**References**	**Sample size**	**Place**	**Scale**	**Standard score (%)**	**Findings**
Edalat Nemoon et al. (22)	186	Urmia	Enrich	26.7	Most of the participants were unemployed (82.5%), had insufficient income (61.5%), lived in cities (79%), and had an academic degree (34.5%). Female factors were the most common cause of infertility among couples (48.5%). There was a significant relationship between marital satisfaction and mental health.
Taghavi et al. (23)	90	Bandar Abbas	Enrich	31.2	Most of the participants had high school education (51.11%). Infertility was the strongest predictor of marital satisfaction in women with polycystic ovary syndrome (*p* < 0.001). Body mass index and husband's age were other significant predictors of marital satisfaction (*p* < 0.05).
Chehreh et al. (21)	200	Tehran	Enrich	60.3	Most of the women had aa academic education (42%). There was a relationship between rejecting a childless lifestyle (one of the dimensions of the fertility problems questionnaire) and marital satisfaction in women (*p* < 0.001).
Mahmoudpour et al. (25)	260	Tehran	Enrich	64.8	Most of the participants were in the age range of 31–40 years (79.09%) and had bachelor's degrees (55.45%). There was a significant negative relationship between incompatible schemas and marital satisfaction (*p* < 0.05).
Mansouri et al. (26)	53	Alborz	Enrich	46.5	Most of the participants had a moderate economic status (84.2%), had a university education (79.3%), and were unemployed (81.5%). There was a significant relationship between women's age and marital satisfaction (*p* < 0.05). Also, the level of marital satisfaction in women whose husbands had more income was higher than in women whose husbands had less income (*P* = 0.024). A significant relationship was found between women's marital satisfaction and education (*P* < 0.001).
Keikhosravi Big Zadeh et al. (24)	225	Tehran	Enrich	62.8	Adaptability to illness scale had a direct and significant relationship with marital satisfaction (*P* < 0.001). There was a direct and significant relationship between marital satisfaction and the scale of compatible strategies of cognitive emotion regulation (*P* < 0.001) and an inverse and significant relationship with the scale of incompatible strategies of cognitive regulation of emotion (*P* < 0.001).
Rajabi et al. (27)	100	Ahvaz	Enrich	60	Most of the participants had undergraduate education (41%). The marital satisfaction score in fertile women was higher than that of infertile women (*p* = 0.05).
Arefi (11)	50	Kermanshah	Enrich	9.4	The average score of marital satisfaction in infertile women was significantly lower than normal women.
Amiri et al. (28)	511	Shahroud	Enrich	25.1	Most participants had primary infertility (71.2%) and were housewives (85.2%). The wife's job (*p* = 0.04), husband's job (*p* = 0.001), and family income (*p* = 0.031) had a significant relationship with marital satisfaction levels.
Sahraian et al. (29)	50	Rasht	Enrich	55	Most of the participants had primary or secondary education (36%), and were unemployed (76%).
					Marital satisfaction in infertile women with female infertility factor and infertile women with male infertility factor had a significant difference (*p* > 0.019). There was a significant positive correlation between the two variables of social support and marital satisfaction.
Shahverdi et al. (30)	100	Kermanshah	Enrich	59.1	Most of the participants had high school education (65%) and were housewives (88%). There was a significant difference between the two groups of fertile and infertile women in terms of marital satisfaction scale (*p* = 0.001).
Zare et al. (12)	110	Mashhad	Nathan Azrin marital satisfaction	79.4	Most participants were housewives (83.6%) with moderate monthly income (82%). There was no significant difference between the two groups of fertile and infertile women regarding marital satisfaction scores (*p* = 0.68). Also, there was a significant relationship between marital satisfaction with education level (*p* < 0.0001) and occupation (*p* = 0.01).
Abedi et al. (31)	138	Sari	Enrich	50.4	Most of the participants living in the city (63.4%) had high school education (38.4%).There was a relationship between the physical dimension of quality of life (and not the psychological dimension) with the marital satisfaction of infertile women
Hatamloye Saedabadi and Hashemi Nosratabad (32)	40	Tabriz	Index of marital satisfaction	41.3	There was no significant difference between the satisfaction scores of the two groups of fertile and infertile women.
Amanelahifard et al. (33)	93	Ahvaz	Enrich	42.6	The marital satisfaction score in infertile women was significantly lower than in fertile women (*p* < 0.001).
Heidari and Latifnejad (34)	125	Mashhad	Enrich	77	Most of the participants had high school education (46.3%), were housewives (7.72%), and had moderate-income (56.3%). The female factor was the most common cause of infertility (41.8%). Marital satisfaction was correlated with anxiety (*p* = 0.003, r = −0.248), self-esteem (*p* = 0.0001, r = 0.328), and social support (*p* = 0.001, r = 0.283).
Bahrainian et al. (35)	90	Tehran	Enrich	44.5	Most participants were between 20 and 30 (91.1%) and had high school education (37.7%). In 63.3% of cases, infertility was due to female factors. Marital satisfaction of fertile women was higher than infertile women.

### Meta-analysis

**Figure 2:**
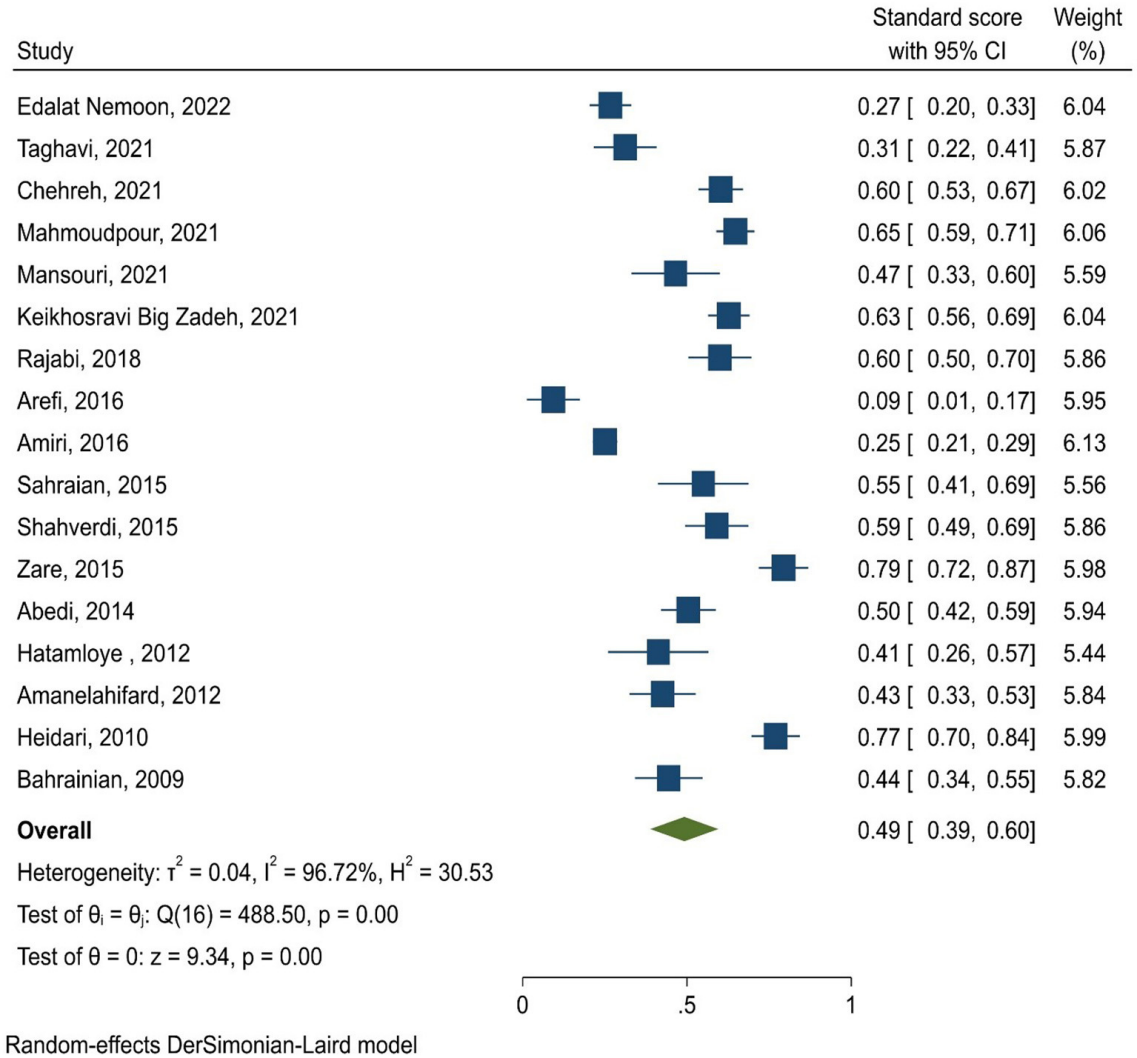
The forest plot of marital satisfaction standard score in infertile women.

**Figure 3:**
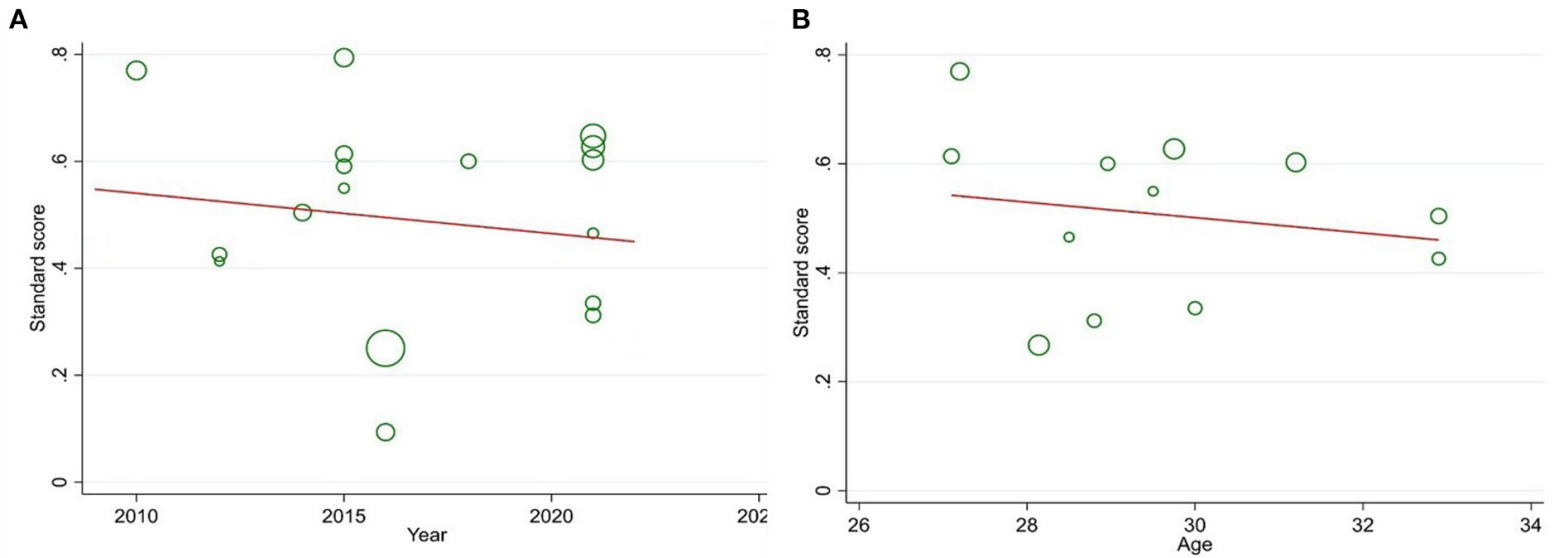
Relationship between year **(A)** and mean age **(B)** with a marital satisfaction standard score.

**Figure 4:**
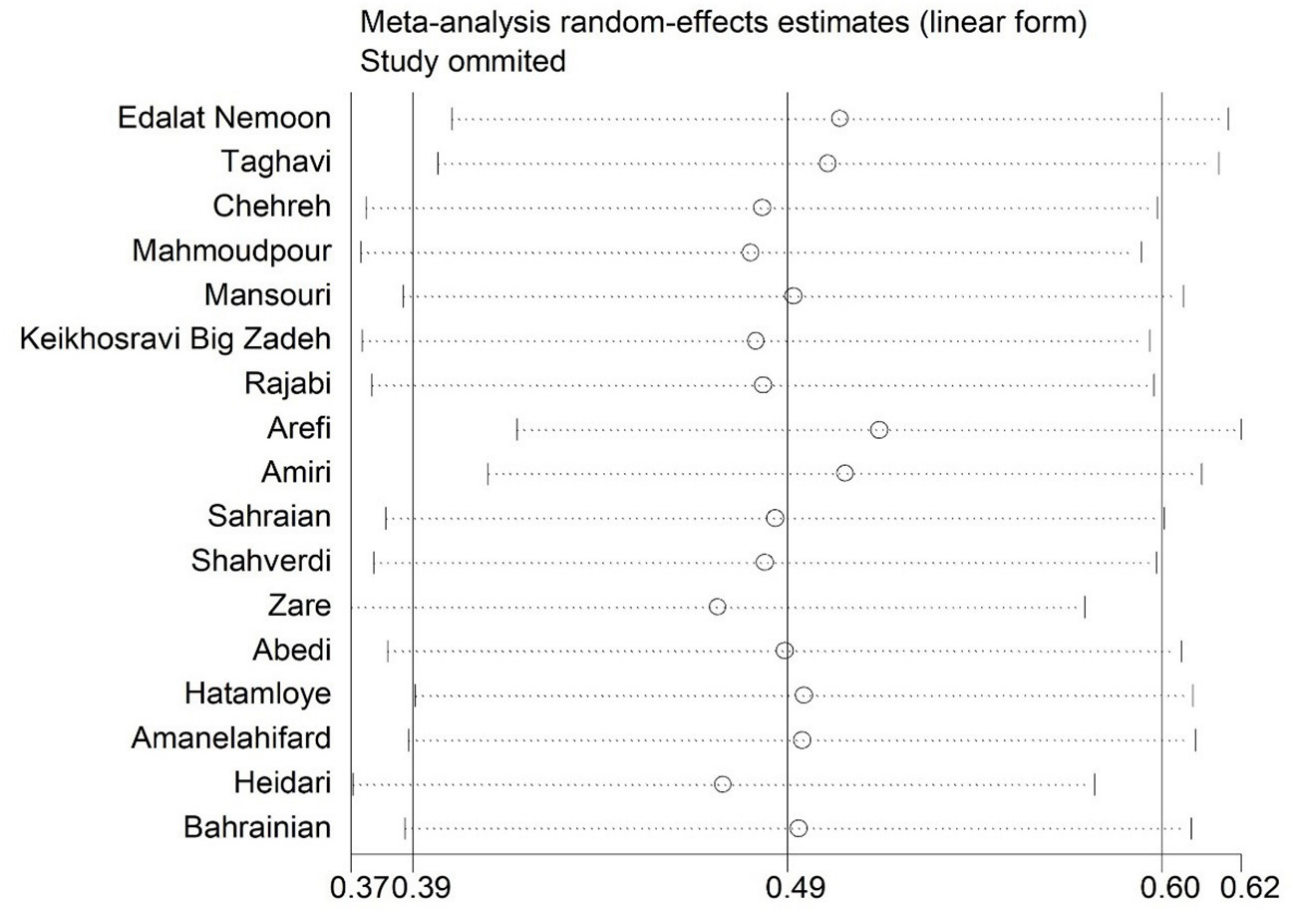
Sensitive analysis.

**Figure 5:**
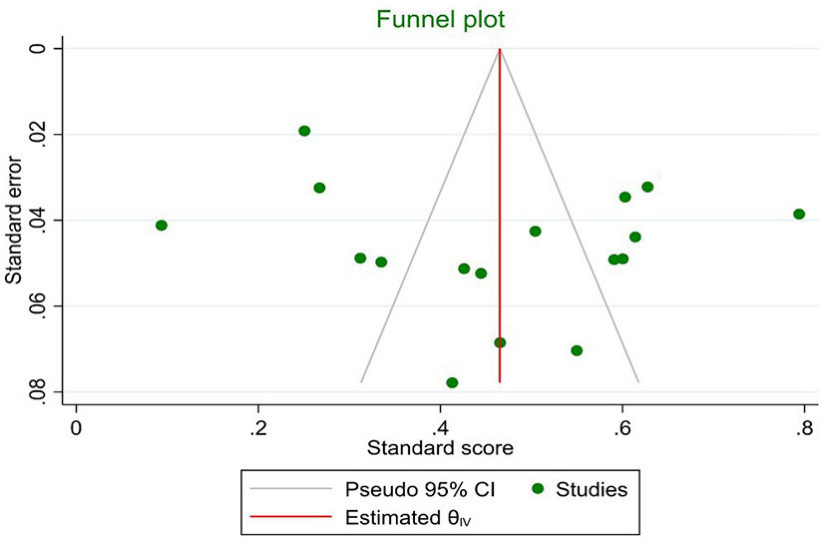
Publication bias.

The marital satisfaction standard score in infertile women was 49% (95% CI: 39–60%) (Figure 2). The pooled standard score of marital satisfaction in the studies conducted in region 1 of the country (Tehran and its neighboring provinces) is 54% (95% CI: 42.7–65.3%), and in other regions of the country was 45% (95% CI: 40–58%). Also, the marital satisfaction pooled score in studies conducted based on the Enrich marital satisfaction scale (15 studies) was 47.7% (95% CI: 37.7–57.7%). In studies conducted with other scales (*n* = 2), it was 56.5% (95% CI: 32.5–80.5%). The meta-regression results indicated that there is no relationship between the standard score of marital satisfaction with the year of the study (*p* = 0.494) and the mean age of infertile women (*p* = 0.580) (Figure 3). The sensitivity analysis showed that by removing each of the studies, the marital satisfaction pooled score would not change (Figure 4). Also, publication bias was insignificant (*p* = 0.904) (Figure 5).

## Discussion

The standard score of marital satisfaction in infertile women was 49%. Because a higher score indicates higher marital satisfaction, the results of this study show that infertile women have moderate to low marital satisfaction. Although infertility affects both sexes equally, women are often blamed (36). They feel guilty, and their self-esteem is threatened. Therefore, they are stigmatized more than men due to infertility and childlessness (37). These women may experience domestic violence, economic deprivation, and ostracized marital lives (10, 38, 39). Infertile women due to social anxiety (such as being sensitive to the opinion of friends and family), relationship anxiety (concern about the effect of infertility on relationships), sexual anxiety (such as decreased sexual pleasure), the need to become a parent, and the rejection of a stress-free lifestyle they experience a lot of psychological problems (40). Since infertile couples often hide this problem and do not share it with their family and relatives, they do not receive support. Over time, they become socially isolated (41).

In addition, infertile women due to social anxiety (such as being sensitive to the opinions of friends and family), relationship anxiety (concern about the effect of infertility on relationships), sexual anxiety (such as decreased sexual pleasure), the need to become parents, and lifestyle rejection. Without children, they experience much psychological stress (40). On the other hand, if relatives learn about the infertility of these women, they may verbally humiliate them. Various studies have shown that relatives used words such as a hollow, fruitless tree, dried tree, and barren land to humiliate these women (36, 42, 43). In Iranian society, the term “silent stove” is used for this couple (44). Another study in Nigeria showed that infertile women are harassed and stigmatized by their husbands' families (45).

Fennel studied couples whose married life lasted more than 20 years. The findings of that study showed that these couples have ten essential characteristics, which are: a lifelong commitment to marriage, commitment to spouse, having strong moral values, respect for one's wife as the best friend, commitment to sexual fidelity, desire to be a good parent, faith in God and spiritual commitment, desire to satisfy and support one's wife, desire to be a good wife and forgive one's wife (46). Infertility can sometimes lead to the collapse of a couple's life by affecting these life-consolidating factors. The results of the study by Tavakol et al. (47) showed that although the presence of children can reduce the time couples spend together, it deepens the relationship of couples. On the other hand, the arrival of children cannot positively affect a marriage already in crisis (47).

The highest marital satisfaction score was related to the studies conducted in Region 1 of the country (Tehran and its neighboring provinces). The reason for this finding can be attributed to the cultural situation of that region. The traditional and patriarchal society in Iran has caused infertility to be considered a kind of stigma, and infertile couples experience much psychological distress in their social interactions, which sometimes causes their lives to fall apart. However, Tehran, as the capital of Iran, has long since become a modern society in which the traditional and patriarchal view has disappeared, so infertile couples suffer less psychological stress in this regard. Slade et al. (48) believe that infertility can have destructive social and psychological consequences (from divorce to social stigma), which cause isolation and psychological distress. There is no relationship between the standard score of marital satisfaction with the year of publication of studies and the average age of infertile women. Considering that the oldest and newest analyzed studies were from 2009 and 2022, it seems that infertile women's marital satisfaction has not changed significantly.

## Strength and limitations of the study

To our knowledge, for the first time, this study has systematically examined the marital satisfaction of infertile women, so these up-to-date results can create a particular perspective for health officials to pay more attention to the mental health indicators of infertile Iranian women. This study had several limitations: Some studies did not report the raw score of marital satisfaction, and in some studies, the marital satisfaction status of infertile couples was investigated, but the scores were not reported separately for men and women. Also, in some studies, the results were misreported. These limitations made it impossible to include these studies in the final analysis.

## Conclusion

This study showed that infertile women have moderate to low marital satisfaction, some of which are rooted in the context and culture of Iranian society. In this society where having children is considered a social value and a necessary condition for married women, childlessness is a deep pain that makes the life of an infertile woman bitter. Therefore, it is necessary for healthcare officials to take measures to increase the marital satisfaction of this group of women (such as holding training classes, providing expert advice and holding discussion sessions).

## Data availability statement

The original contributions presented in the study are included in the article/supplementary material, further inquiries can be directed to the corresponding author/s.

## Author contributions

AT and RG contributed to the design, writing, editing the paper, and performing of this systematic review. AF, VB, and BS checked the data and conducted data analyses. All authors read and confirmed the final version of the manuscript.

## Conflict of interest

The authors declare that the research was conducted in the absence of any commercial or financial relationships that could be construed as a potential conflict of interest.

## Publisher's note

All claims expressed in this article are solely those of the authors and do not necessarily represent those of their affiliated organizations, or those of the publisher, the editors and the reviewers. Any product that may be evaluated in this article, or claim that may be made by its manufacturer, is not guaranteed or endorsed by the publisher.
